# Relative Contributions of Prenylation and Postprenylation Processing in *Cryptococcus neoformans* Pathogenesis

**DOI:** 10.1128/mSphere.00084-15

**Published:** 2016-03-30

**Authors:** Shannon K. Esher, Kyla S. Ost, Lukasz Kozubowski, Dong-Hoon Yang, Min Su Kim, Yong-Sun Bahn, J. Andrew Alspaugh, Connie B. Nichols

**Affiliations:** a Departments of Molecular Genetics and Microbiology/Medicine, Duke University School of Medicine, Durham, North Carolina, USA; b Department of Genetics and Biochemistry, Clemson University, Clemson, South Carolina, USA; c Department of Biotechnology, College of Life Science and Biotechnology, Yonsei University, Seoul, Republic of Korea; Carnegie Mellon University

**Keywords:** *Cryptococcus neoformans*, farnesyltransferase, fungal pathogen, protein prenylation, Ras-like GTPases

## Abstract

Prenyltransferase enzymes promote the membrane localization of their target proteins by directing the attachment of a hydrophobic lipid group at a conserved C-terminal CAAX motif. Subsequently, the prenylated protein is further modified by postprenylation processing enzymes that cleave the terminal 3 amino acids and carboxymethylate the prenylated cysteine residue. Many prenylated proteins, including Ras1 and Ras-like proteins, require this multistep membrane localization process in order to function properly. In the human fungal pathogen *Cryptococcus neoformans*, previous studies have demonstrated that two distinct forms of protein prenylation, farnesylation and geranylgeranylation, are both required for cellular adaptation to stress, as well as full virulence in animal infection models. Here, we establish that the *C. neoformans RAM1* gene encoding the farnesyltransferase β-subunit, though not strictly essential for growth under permissive *in vitro* conditions, is absolutely required for cryptococcal pathogenesis. We also identify and characterize postprenylation protease and carboxyl methyltransferase enzymes in *C. neoformans*. In contrast to the prenyltransferases, deletion of the genes encoding the Rce1 protease and Ste14 carboxyl methyltransferase results in subtle defects in stress response and only partial reductions in virulence. These postprenylation modifications, as well as the prenylation events themselves, do play important roles in mating and hyphal transitions, likely due to their regulation of peptide pheromones and other proteins involved in development.

**IMPORTANCE** *Cryptococcus neoformans* is an important human fungal pathogen that causes disease and death in immunocompromised individuals. The growth and morphogenesis of this fungus are controlled by conserved Ras-like GTPases, which are also important for its pathogenicity. Many of these proteins require proper subcellular localization for full function, and they are directed to cellular membranes through a posttranslational modification process known as prenylation. These studies investigate the roles of one of the prenylation enzymes, farnesyltransferase, as well as the postprenylation processing enzymes in *C. neoformans*. We demonstrate that the postprenylation processing steps are dispensable for the localization of certain substrate proteins. However, both protein farnesylation and the subsequent postprenylation processing steps are required for full pathogenesis of this fungus.

## Abstract (precis)

*Cryptococcus neoformans* is an important human fungal pathogen that causes disease and death in immunocompromised individuals. The growth and morphogenesis of this fungus are controlled by conserved Ras-like GTPases, which are also important for its pathogenicity. Many of these proteins require proper subcellular localization for full function, and they are directed to cellular membranes through a posttranslational modification process known as prenylation. These studies investigate the roles of one of the prenylation enzymes, farnesyltransferase, as well as the postprenylation processing enzymes in *C. neoformans*. We demonstrate that the postprenylation processing steps are dispensable for the localization of certain substrate proteins. However, both protein farnesylation and the subsequent postprenylation processing steps are required for full pathogenesis of this fungus.

## INTRODUCTION

Ras-like GTPases are important for the growth and morphogenesis of many small eukaryotes, such as fungi (1–7). For full function, these proteins require proper subcellular localization, often to various cellular membranes (8–13). Many Ras-like GTPase proteins are directed to the membrane by prenylation, a process of posttranslational modification involving the covalent attachment of a lipid moiety to a cysteine residue near the C terminus of a substrate protein. This process occurs in the cytoplasm and is irreversible (14–16).

Prenylation substrates are characterized by their C-terminal CAAX motif, where C is the modified cysteine, A represents an aliphatic amino acid, and X represents a variable amino acid. Prenylation of CAAX substrates is mediated by either farnesyltransferase (FTase) or geranylgeranyltransferase I (GGTase-I), which catalyze the addition of the 15-carbon or 20-carbon lipid group, respectively. The prenyltransferase enzymes are heterodimers composed of a common α-subunit and distinct β-subunits that mediate target specificity and differential prenyl group addition. Classically, the amino acid in the X position confers substrate specificity, with proteins terminating in glutamine, methionine, or serine being preferentially processed by FTase and proteins terminating in leucine most often processed by GGTase-I (15–17). However, recent evidence suggests overlapping substrate utilization and cross-specificity between these enzymes (8, 14, 15, 18).

Following prenylation by either FTase or GGTase-I, prenylated substrates undergo postprenylation processing steps to form the fully mature protein. First the terminal –AAX motif is endoproteolytically cleaved. Two CAAX proteases, Ras processing enzyme 1 (Rce1) and Ste24, have been identified in *Saccharomyces cerevisiae* and in higher eukaryotes, including humans (RCE1 and ZMPSTE24). Similar to prenylation, this step is irreversible. Subsequently, the prenylated cysteine is carboxymethylated by the enzyme isoprenylcysteine carboxyl methyltransferase (ICMT), known as Ste14 in *S. cerevisiae*. Carboxyl methylation cannot occur without cleavage of the terminal –AAX. Studies have shown that prenylation and postprenylation processing enzymes localize and function within the endoplasmic reticulum (ER) (19, 20).

Due to the role of Ras and other small GTPases in oncogenesis, the prenylation pathway has been extensively studied for therapeutic targeting. A number of FTase inhibitors (FTIs) have been developed to potentially disrupt the localization and function of Ras-like proteins and other prenylated substrates. A common issue that arises is the potential for cross-prenylation by GGTase-I in the absence of FTase (21–24). To address this possibility, the postprenylation steps of CAAX motif cleavage and carboxyl methylation have also been investigated as therapeutic targets. Since both farnesylated and geranylgeranylated proteins are processed by these downstream enzymes, targeting postprenylation modifying enzymes has the potential to overcome both issues of prenyltransferase substrate specificity and cross-prenylation (23–25).

Ras family proteins play crucial roles in cellular growth and morphogenesis in a number of human microbial pathogens. Furthermore, the functions of these proteins, including their roles in virulence, have been shown to be dependent on their subcellular localization, in part due to their prenylation (2). For example, in the pathogenic fungus *Candida albicans*, the Ras1 protein requires proper membrane localization to support hyphal growth, an important virulence-associated phenotype of this organism (11). In *Aspergillus fumigatus*, mislocalization of RasA from the plasma membrane (PM) results in impaired hyphal growth, defective cell walls, and decreased virulence (12). Components of the prenylation pathway, in particular the prenyltransferases, have also been shown to play important roles in fungal virulence. The *C. albicans* RAM2 gene, encoding the shared α-subunit of FTase and GGTase-I, is essential for viability (26). Similarly, *RAM2* expression in *Candida glabrata* is required for viability both *in vitro* and *in vivo* within infected mice (27).

We have previously demonstrated the importance of both FTase and GGTase-I in the growth and virulence of the human-pathogenic fungus *Cryptococcus neoformans* (8, 28). *C. neoformans* causes significant disease and death in immunocompromised patients, estimated to result in over 600,000 deaths each year in patients infected with HIV (29). Ras-like GTPases have been identified and characterized extensively in *C. neoformans* (6, 7, 9, 28, 30–33). Furthermore, the role of prenylation in the function of these proteins has also been demonstrated (34, 35). CnRas1, which is required for thermotolerance, morphogenesis, and mating, is unable to support these cellular functions when its CAAX motif is disrupted (8, 9). Similarly the Cdc42 GTPase, which is important for high-temperature growth and actin/septin organization during morphogenesis, requires prenylation-dependent membrane localization for full activity (8, 36). In previous studies, Cdc42 has been shown to be a GGTase-I substrate: membrane localization of this GTPase is disrupted in strains lacking the β-subunit of GGTase-I, Cdc43 (8). However, while Ras1 and the Rac2 GTPase are predicted GGTase-I substrates based on their CAAX motifs, neither protein is mislocalized in the *cdc43*Δ GGTase-I mutant, suggesting that they may be FTase substrates (8). Importantly, the roles of the downstream prenylation pathway members (Rce1, Ste24, and Ste14) in the localization of these small GTPases have not yet been defined in *C. neoformans*.

To examine the prenylation and postprenylation processing pathways of *C. neoformans*, we have identified and characterized genes encoding the proteins predicted to be involved in each step of this process. We have examined their individual roles in the growth, morphogenesis, and virulence of *C. neoformans* and characterized their impact on protein localization. Our studies reveal that the initial step of prenylation, mediated by prenyltransferases, is required for the proper localization and function of Ras-like GTPase function. Moreover, these enzymes are absolutely required for survival of the pathogen *in vivo*. However, in comparison, we have demonstrated that the postprenylation processing enzymes are not required for Ras1 GTPase membrane localization while still mediating a role in fungal pathogenesis.

## RESULTS

### Mutation of the *C. neoformans* FTase β-subunit *RAM1* gene.

Previously, we identified and characterized *RAM1*, the gene encoding the *β*-subunit of FTase in *C. neoformans* var. *neoformans* (35). In this variety of *C. neoformans*, we were unable to isolate a haploid *ram1*Δ mutant from recombinant spores of a diploid, heterozygous *RAM1*/*ram1* mutant strain. We therefore concluded that *RAM1* was essential under the specific *in vitro* conditions tested (35). However, based on data from prenylation in other systems, we hypothesized that *RAM1* may not be an essential gene under all conditions and that we might be able to generate a *ram1*Δ mutant in the fully virulent *C. neoformans* var. *grubii* strain background by using a mutant strain (*cku80* mutant) with impaired nonhomologous end joining and enhanced homologous recombination, as well as using very permissive growth conditions (37–39). Using a BLAST search with the *C. neoformans* var. *neoformans RAM1* gene (35) as a query, we identified CNAG_05740 as the gene encoding Ram1 in the *C. neoformans* var. *grubii* genome database (H99 Sequencing Project, Broad Institute of Harvard and MIT). We generated several strains with a targeted deletion of the CNAG_05740 locus in the *cku80*Δ mutant strain background, replacing the entire open reading frame (ORF) with the neomycin-resistant dominant selectable marker (40). Transformants were incubated at 25°C to enrich for mutants with potentially impaired stress tolerance. In this manner, we isolated 9 *ram1*Δ mutants among 10 transformants. Once we confirmed that *RAM1* was not absolutely essential for viability in the *cku80* strain background, we generated *ram1*Δ mutant strains in the wild-type (WT) strain (H99) to use in subsequent experiments.

### Ram1 is required for high-temperature growth.

**FIG 1:**
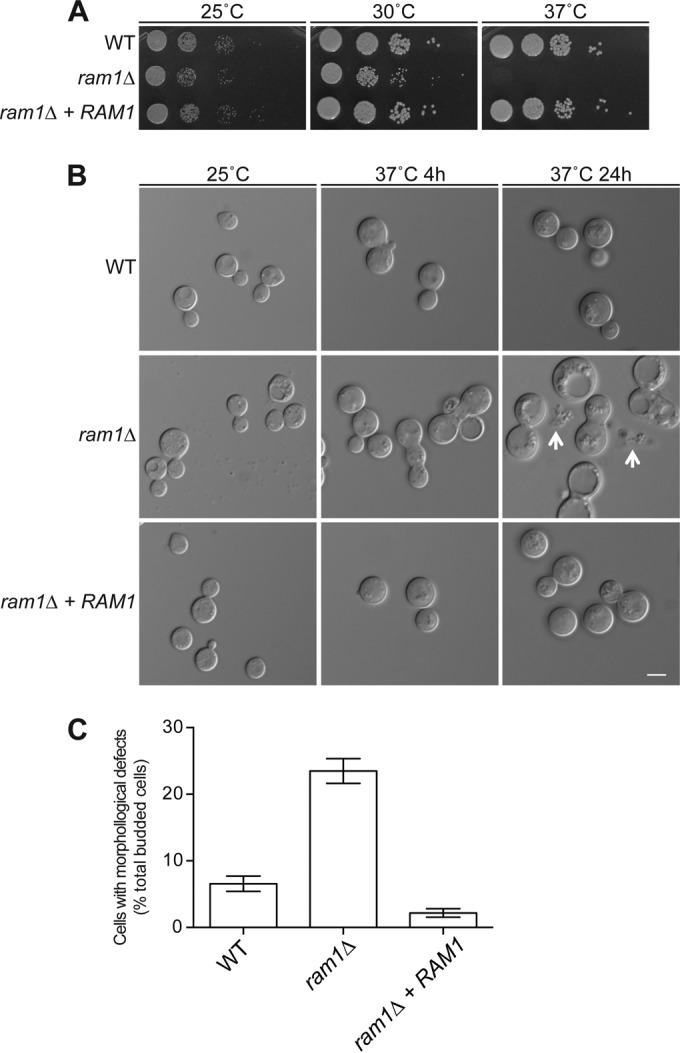
*C. neoformans* Ram1 is required for thermotolerance. (A) Overnight cultures of the wild-type (H99), *ram1*Δ (SKE1), and *ram1*Δ + *RAM1* reconstituted (SKE39) strains were serially diluted, spotted onto YPD medium, and incubated at 25°C, 30°C, and 37°C for 48 h. (B) The wild-type (H99), *ram1*Δ (SKE1), and *ram1*Δ + *RAM1* (SKE39) strains were incubated until reaching mid-logarithmic-phase growth at 25°C, diluted, and shifted to either 25°C or 37°C for 4 h. Cells were imaged with DIC optics to assess temperature-dependent alterations in morphology. Arrows indicate evidence of cell lysis. Bar, 5 µm. (C) Quantification of morphological defects after 4 h of incubation at 37°C. Data represent the percentages (as means ± standard errors) of budded cells with morphological defects, which included wide bud necks and/or chains of unbudded cells. At least 400 cells over 3 biological replicates of each strain were counted using ImageJ software (Fiji) (60). *P* < 0.0001 for *ram1*Δ mutant versus WT and *ram1*Δ mutant versus *ram1*Δ + *RAM1* mutant as determined by one-way ANOVA and Tukey’s multiple comparisons test.

The ability to grow at 37°C is essential for the pathogenicity of *C. neoformans*, and we previously demonstrated that several targets of prenylation are required for thermotolerance in this pathogen (6, 8, 9, 32). The *ram1*Δ mutant exhibited a growth defect compared to the wild type at 30°C, with all growth abolished at 37°C (Fig. 1A). This phenotype was complemented in a reconstituted strain in which the wild-type *RAM1* gene was reintroduced. We did not observe defects in these strains for capsule or melanin production, two well-characterized virulence factors (data not shown).

To further elucidate the temperature sensitivity of the *ram1*Δ mutant strain, we microscopically examined cell morphology at various incubation temperatures. Previous work demonstrated temperature-dependent morphological defects associated with mutations in *C. neoformans* prenylated proteins (7, 32). Microscopic analysis performed at serial time points revealed that *ram1*Δ mutant cells incubated at 25°C displayed a cell size and morphology similar to those of the wild-type and reconstituted strains. However, after 4 h of incubation at 37°C, we observed that 23.5% ± 1.85% of the *ram1*Δ mutant cells (compared to 6.6% ± 1.2% of WT cells and 2.2% ± 0.6% of *ram1Δ + RAM1* reconstituted cells) displayed morphological changes, including wide bud necks and chains of unseparated budding cells, consistent with a defect in cytokinesis. By 24 h, the *ram1*Δ mutant cells displayed marked cytokinesis defects and evidence of cell lysis in the majority of cells in the culture (Fig. 1B and C). These changes were absent in the reconstituted strain, suggesting that the *RAM1* mutation was responsible for the temperature-dependent morphogenesis defects.

### Ram1 is required for full Ras1 membrane localization.

**FIG 2:**
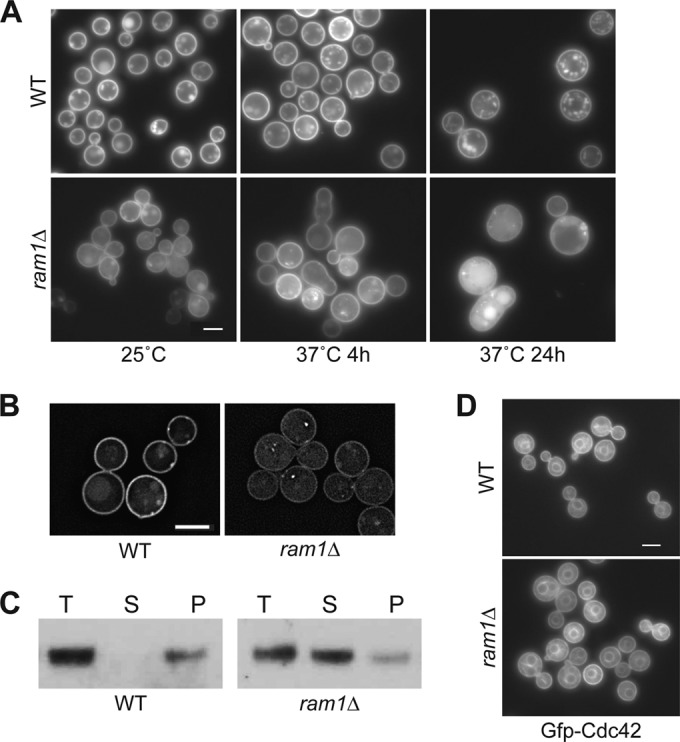
Ras1 is a specific target of Ram1. (A and B) Ras1 localization to the PM is reduced in the *ram1*Δ mutant strain. To compare the patterns and intensities of mCherry-Ras1 fusion protein localization, wild-type (CBN121) and *ram1*Δ mutant (SKE17) strains expressing *mCh-RAS1* under the control of the histone H3 promoter were incubated in rich medium, diluted 10-fold, and subsequently incubated for 4 or 24 h at 25°C and 37°C. (A) Live cells grown at 25°C or 37°C for 4 h and at 37°C for 24 h were imaged by fluorescence microscopy (Zeiss Axio Imager A1) using the appropriate filter. Bar, 5 µm. (B) Live cells incubated at 25°C for 4 h were also imaged using DeltaVision deconvolution microscopy with the appropriate filter. Images were deconvolved using softWoRx software. Bar, 5 µm. Images were taken using identical exposures. (C) Ras1 protein accumulates in the cytoplasm in the *ram1*Δ mutant strain. To compare the relative amounts of mCherry-Ras1 protein on cellular membranes and in the cytoplasm, total lysates from strains CBN121 and SKE17 were separated into soluble (cytoplasmic) and insoluble (membrane-associated) fractions by ultracentrifugation. Total lysate (T), soluble (S), and insoluble pellet (P) samples were assessed by Western blotting with an antibody specific to the mCherry fusion protein. (D) Cdc42 localization is not dependent on Ram1. Wild-type (CBN302) and *ram1*Δ mutant (SKE19) strains expressing *GFP-CDC42* under the control of the histone H3 promoter were incubated overnight in YPD, diluted 10-fold, and grown for 4 h at 25°C. Live cells were imaged (Zeiss Axio Imager A1) using the appropriate filter. Bar, 5 µm.

Since the *C. neoformans* Ras1 protein requires prenylation-dependent membrane localization to be fully functional (9), we hypothesized that this localization would be altered in the *ram1*Δ mutant strain, perhaps contributing to some of the observed morphological defects. To test this hypothesis, we examined the subcellular localization pattern of a constitutively expressed, fluorescently tagged Ras1 protein in the wild-type (H99) and the *ram1*Δ mutant strains (Fig. 2A). Consistent with previous data, mCherry-Ras1 (mCh-Ras1) localized primarily to the PM in wild-type cells when incubated either at 25°C or 37°C. In contrast, in the *ram1*Δ mutant strain, we detected a shift of localization of mCh-Ras1 from the PM to the cytoplasm in cells incubated at 25°C, and the shift was more pronounced in cells incubated at 37°C for 4 h and 24 h. We further explored the altered localization of Ras1 in the *ram1*Δ mutant strain by two methods. First, we visualized mCh-Ras1 localization using high-resolution microscopy and similarly observed an increased mCh-Ras1 signal in the cytoplasm and a decreased signal at the PM in the *ram1*Δ mutant, compared to strong PM localization in wild-type cells (Fig. 2B). Second, we confirmed this observation by comparing Ras1 protein levels in the soluble (cytoplasmic) and insoluble (membrane-associated) fractions of wild-type and *ram1*Δ mutant strains expressing mCh-Ras1 by using subcellular fractionation and Western blot analysis (Fig. 2C). In wild-type cells, mCh-Ras1 was detected only in the insoluble pellet fraction, consistent with the observed PM localization of mCh-Ras1. In contrast, in the *ram1*Δ mutant strain, mCh-Ras1 protein was detected in the cytoplasmic/soluble fraction in addition to the control (total lysate) and insoluble pellet fractions. This result demonstrates that Ras1 is associated with the cytoplasm in the *ram1*Δ mutant strain and confirms our fluorescence localization results, suggesting that the localization of Ras1 is shifted from the PM to the cytoplasm in the absence of the Ram1 FTase.

We also examined the impact of the Ram1 mutation on the localization of the Cdc42 protein, which we previously demonstrated to be dependent on the GGTase-I Cdc43 (8). Consistent with prior observations, the green fluorescent protein (Gfp)-Cdc42 fusion protein localized primarily to the PM and endomembranes in wild-type cells (Fig. 2D) (8, 41). Gfp-Cdc42 localization was unchanged in the *ram1*Δ mutant strain, confirming that Cdc42 is primarily a substrate of GGTase-I rather than FTase. Although there may be cross-prenylation of certain proteins, these results demonstrate that at least some prenylated *C. neoformans* proteins are specifically targeted by either FTase or GGTase-I.

### Identification of postprenylation pathway components in *C. neoformans*

To characterize the contributions of components of the postprenylation processing pathway in *C. neoformans*, we identified and deleted genes predicted to encode enzymes involved in the serial posttranslational processing events that occur after prenylation. We identified predicted orthologs of the Rce1 and Ste24 CAAX proteases (CNAG_01978 and CNAG_ 01378, respectively) and the Ste14 carboxyl methyltransferase (CNAG_01592) in the *C. neoformans* var. *grubii* genome database by using the *S. cerevisiae* protein homologs as queries. Single predicted orthologs for each gene were identified in the *C. neoformans* genome, and deletion strains for each gene were generated using homologous recombination, replacing each ORF with a dominant selectable marker**.** We also constructed a double *rce1Δ ste24*Δ protease mutant, given the potential for functional redundancy.

**FIG 3:**
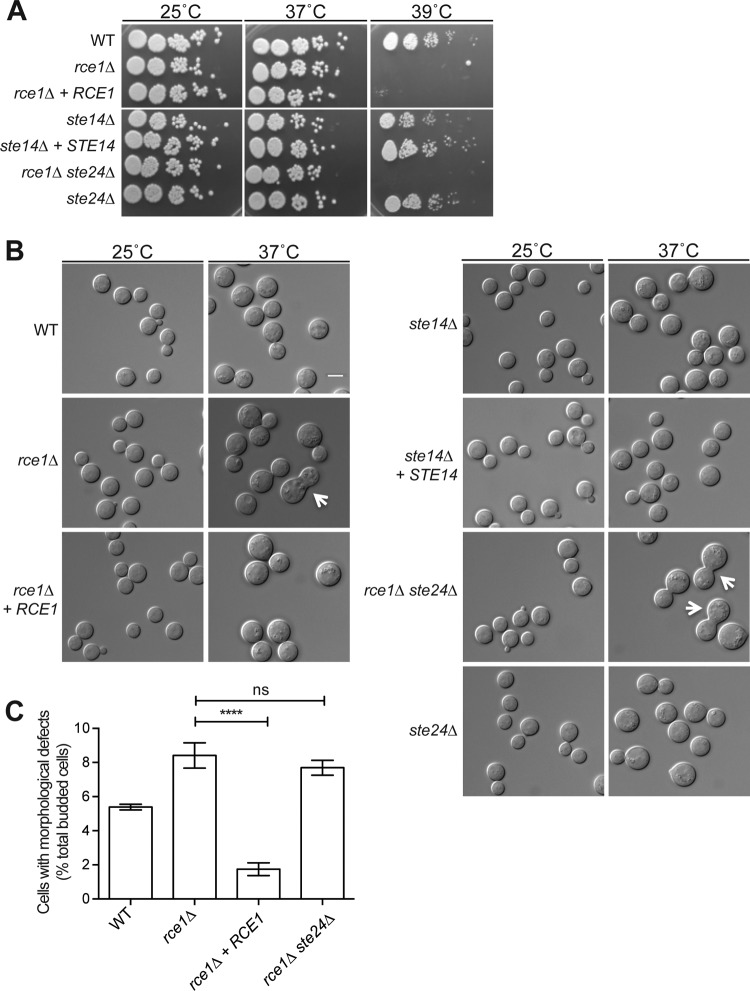
Phenotypic analysis of the postprenylation processing mutant strains. (A) The *rce1*Δ protease mutant is temperature sensitive. The wild-type (H99), *rce1*Δ (LKA1), *rce1*Δ + *RCE1* reconstituted (SKE30), *ste14*Δ (YSB1187), ste14Δ + *STE14* reconstituted (YSB1597), *ste24*Δ (LKA14), and *rce1Δ ste24*Δ double mutant (LKA18) strains were serially diluted, spotted onto YPD medium, and incubated at 25°C, 37°C, and 39°C for 48 h. (B and C) The *rce1*Δ protease mutant strain exhibits a temperature-dependent cytokinesis defect. (B) The wild-type (H99), *rce1*Δ (LKA1), *rce1*Δ + *RCE1* reconstituted (SKE30), *ste14*Δ (YSB1187), ste14Δ + *STE14* reconstituted (YSB1597), *ste24*Δ (LKA14), and *rce1Δ ste24*Δ double mutant (LKA18) strains were incubated in YPD liquid medium and shifted to either 25°C or 37°C for 24 h. Cells were imaged with DIC optics to assess temperature-dependent alterations in morphology. Arrows denote wide bud necks indicating the partial cytokinesis defects of the *rce1*Δ and *rce1Δ ste24*Δ mutant strains. Bar, 5 µm. (C) Quantification of morphological defects after 24 h of incubation at 37°C. Data represent the percentages (as means ± standard errors) of budded cells with morphological defects (wide bud necks, elongated/unseparated cells, and/or chains of cells). At least 600 cells over 3 biological replicates of each strain were counted using ImageJ software (Fiji) (60). Statistical significance was determined by one-way ANOVA and Tukey’s multiple comparisons test. ****, *P* < 0.0001 (*rce1*Δ mutant versus *rce1*Δ + *RCE1* mutant); ns, not significant (*rce1*Δ mutant versus *rce1Δ ste24*Δ mutant).

In contrast to the *ram1*Δ mutant strain, the predicted postprenylation processing genes had more subtle defects in thermotolerance (Fig. 3A). Each of the predicted downstream prenylation processing mutant strains (*rce1*Δ, *ste14*Δ, *ste24*Δ, and *rce1Δ ste24*Δ mutants) grew comparably to the wild type at 30°C and 37°C. The *rce1*Δ CAAX protease mutant and the *rce1Δ ste24*Δ double mutant strains displayed notable growth defects when incubated at 39°C. The *ste14*Δ strain (carboxymethyltransferase mutant) demonstrated a more subtle growth impairment at 39°C, manifested as a smaller colony size compared to that of the wild type (Fig. 3A).

We noted partial suppression of the high-temperature growth defect of the *rce1*Δ mutant in the *rce1*Δ + *RCE1* reconstituted strain. To ensure that the temperature-sensitive growth defect was in fact associated with the *RCE1* mutations, we created an independent *rce1*Δ mutant (strain SKE51 [*rce1*Δ^b^]). Compared to the original mutant (strain LKA1 [*rce1*Δ^a^]), this strain demonstrated a similar growth defect at 39°C (see Fig. S2A in the supplemental material).

In addition to assessing thermotolerance, we microscopically examined individual cells for temperature-dependent morphological and cytokinesis defects suggestive of defective function of prenylated proteins. When incubated at 37°C, 8.4% ± 0.7% of the *rce1*Δ mutant strain cells displayed morphological changes, including elongated/unbudded cells and wide bud necks, compared to 5.4% ± 0.2% of WT cells (Fig. 3B and C). The morphological changes were similar to though not as severe as those in the *ram1*Δ mutant. All morphology defects were completely restored in the *rce1*Δ + *RCE1* reconstituted strain (1.7% ± 0.4%). Additionally, similar temperature-dependent morphological changes were observed in the two independently derived *rce1*Δ mutants (LKA1 [*rce1Δ^a^*] and SKE51 [*rce1Δ^b^*]) (see Fig. S2B and C in the supplemental material). The *ste24*Δ mutant strain exhibited normal morphology at 25°C and 37°C, and there was no additive morphological phenotype in the double *rce1Δ ste24*Δ double mutant (7.7% ± 0.4%) compared to the phenotype of the single *rce1*Δ mutant strain, suggesting that Ste24 does not contribute significantly to budding and cytokinesis in response to temperature stress. Consistent with its less prominent defect in thermotolerance, we did not observe any temperature-dependent morphological changes in the *ste14*Δ mutant strain (Fig. 3B). Together, these results suggest that the predicted postprenylation processing enzymes are collectively important for efficient cytokinesis during cell stress, though not for thermotolerance at 37°C.

### Postprenylation processing does not affect Ras1 or Cdc42 membrane localization.

**FIG 4:**
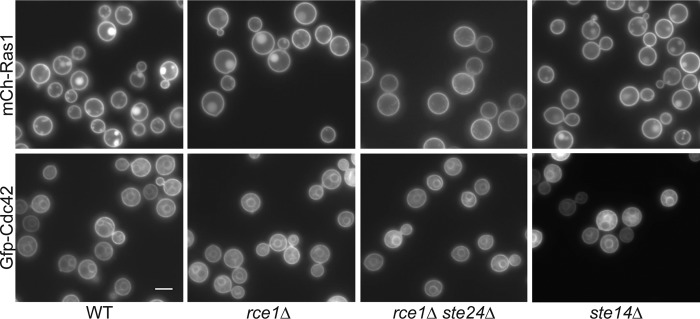
Localization of prenylation substrates Ras1 and Cdc42 is not impaired by loss of the postprenylation processing enzymes. To compare the membrane localization of Ras1 and Cdc42 in the postprenylation processing mutants, wild-type and mutant strains expressing *mCh-RAS1* (top) or *GFP-CDC42* (bottom) were incubated overnight in YPD, diluted 10-fold, and grown for 4 h at 25°C. (Top) mCh-Ras1 expression in wild-type (CBN121), *rce1*Δ (LKA7), *rce1Δ ste24*Δ (CBN48), and *ste14*Δ (CBN308) strains. (Bottom) Gfp-Cdc42 expression in wild-type (CBN302), *rce1*Δ (SKE15), *rce1Δ ste24*Δ (SKE22), and *ste14*Δ (SKE53) strains. Cells were imaged by fluorescence microscopy (Zeiss Axio Imager A1) using the appropriate filter. Bar, 5 µm.

Since prenylated proteins like Ras1 and Cdc42 require membrane localization to be fully functional (9), we hypothesized that the proper membrane localization of these important GTPases may be altered in postprenylation mutant strains. We examined the localization of mCh-Ras1 and Gfp-Cdc42 in the *rce1*Δ, *rce1Δ ste24*Δ, and *ste14*Δ mutant strains (Fig. 4). As described previously, in wild-type cells, mCh-Ras1 localized to the PM, while Gfp-Cdc42 localized to the PM and endomembrane structures. Interestingly, mutation of the terminal prenylation processing proteins did not noticeably affect either Ras1 or Cdc42 membrane localization. These observations suggest that, while prenylation itself is required for target protein membrane localization, the postprenylation protein processing events are not individually required for the localization and function of these prenylated proteins in *C. neoformans*.

### The prenylation pathway plays a role in *C. neoformans* sexual reproduction.

**FIG 5:**
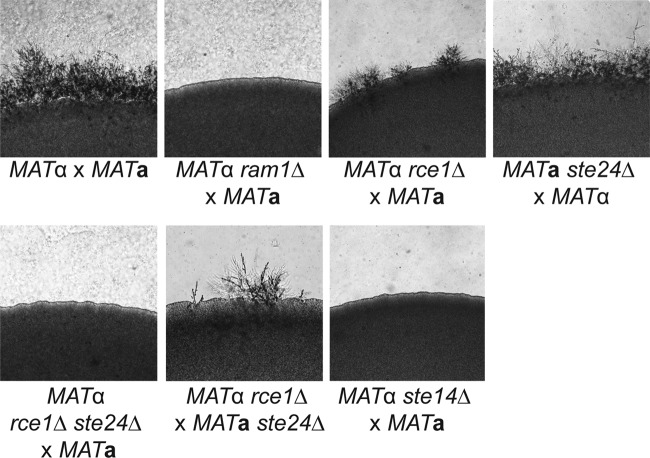
Prenylation and postprocessing enzymes Ram1, Rce1, Ste24, and Ste14 play a role in mating filament formation during *C. neoformans* sexual development. Overnight cultures of *MAT*α wild-type (H99) and *ram1*Δ (SKE1), *rce1*Δ (LKA1), *rce1Δ ste24*Δ (LKA18), and *ste14*Δ (YSB1187) mutant strains were each mixed with equal amounts of the wild-type *MAT***a** strain (KN99**a**). Similarly, the *MAT***a** *ste24*Δ strain (LKA14) was mixed with equal amounts of the *MAT*α wild-type (H99) and *MAT*α *rce1*Δ (LKA1) strains. Each mating mixture was spotted onto MS mating medium and incubated for 7 days in the dark. Images were taken at the peripheries of the mating mixtures.

In *C. neoformans*, multiple proteins involved in sexual reproduction are predicted to be prenylated, including Ras1 and the MF-alpha 1 pheromone (6, 35, 42). In addition, the GGTase-I mutant *cdc43*Δ strain exhibits a temporal delay in mating filament production (8). We assessed the mating competence of the *ram1*Δ mutant strain and the postprenylation processing mutant strains in simple unilateral mating assays in which each mutant strain was cocultured on MS mating medium with a wild-type mating partner of the opposite mating type (Fig. 5). In this assay, we found that the wild-type control mating reaction (*MAT*α H99 × *MAT***a** KN99**a**) produced abundant filaments after 1 week of incubation. We were unable to detect any mating filaments in the *ram1*Δ or *ste14*Δ mutant mating reaction cultures (*ram1*Δ × KN99**a** and *ste14*Δ × KN99**a**), suggesting that these mutants are completely sterile. The *rce1*Δ protease mutant strain also demonstrated a deficiency in mating. In these assays, mating was reduced to patchy, isolated foci. This reduction in mating was also observed when the *rce1*Δ strain was crossed to a *ste24*Δ mutant strain (*MAT*α *rce1*Δ × *MAT***a** *ste24*Δ). However, the *ste24*Δ protease mutant strain did not exhibit any defects in unilateral mating reactions (*MAT*α H99 × *MAT***a** *ste24*Δ). Although reduced in mating filament production, the protease mutant strain crosses produced filaments, basidia, and basidiospores indistinguishable from those resulting from the wild-type mating reaction. Interestingly, although neither protease single mutant strain was sterile, the *rce1Δ ste24*Δ double protease mutant strain was completely sterile, unable to produce any mating filaments when crossed to the wild type (*rce1Δ ste24*Δ × KN99**a**). These results demonstrate that the Ram1 FTase subunit and the Ste14 protein are absolutely required for the cryptococcal mating process. They also suggest that the Rce1 and Ste24 proteases play overlapping roles in *C. neoformans* sexual differentiation, though Rce1 is likely the more important CAAX protease for mating.

### Virulence effects of farnesylation and postprenylation processing.

**FIG 6:**
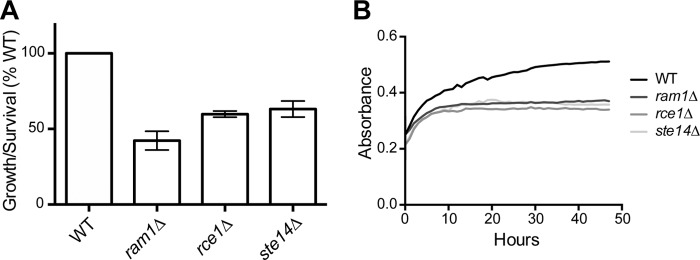
*In vitro* virulence assessment of farnesylation and postprenylation processing enzymes. (A) The *ram1*Δ, *rce1*Δ, and *ste14*Δ mutant strains display decreased survival in macrophages. Wild-type H99 and *ram1*Δ (SKE1), *rce1*Δ (SKE51), and *ste14*Δ (YSB1187) mutant strains were each coincubated with activated J774.1 cells for 1 h, followed by removal of nonphagocytosed yeast cells. Cryptococcal cell survival was assessed at 24 h by quantitative culture. Data represent mean results ± standard errors from 4 replicates for each WT-mutant pair. *P* < 0.05 for all strains versus WT, as determined by one-way ANOVA. (B) The *ram1*Δ, *rce1*Δ, and *ste14*Δ mutant strains have growth defects in DMEM macrophage medium. Wild-type H99 and *ram1*Δ (SKE1), *rce1*Δ (SKE51), and *ste14*Δ (YSB1187) mutant strains were incubated overnight in YPD, normalized and diluted into DMEM, and plated in a 96-well plate. Cells were grown for 48 h in a microplate reader, with absorbance readings taken every hour.

Due to the severe thermotolerance defect of the *C. neoformans ram1*Δ mutant, we hypothesized that this strain would have a similarly notable defect in virulence. To address this, we first examined the intracellular survival of the *ram1*Δ mutant strain in coculture experiments with J774.1 murine macrophages (43). We also included the *rce1*Δ and *ste14*Δ mutant strains, given their thermotolerance growth defects, to determine whether the deletion of either of these genes would affect virulence in *C. neoformans*. Compared to the results for the wild type, we found that each mutant strain exhibited a significant decrease in growth/survival in this assay. The total number of fungal cells remaining viable after 24 h of coincubation with activated J774.1 cells was expressed as the ratio to the total number of *C. neoformans* cells added at the beginning of the experiment (output/input ratio). The *ram1*Δ, *rce1*Δ, and *ste14*Δ mutant strains displayed significantly reduced survival in the macrophage coculture (output-input ratios of 42%, 55%, and 63%, respectively) compared to the survival of the wild type (Fig. 6A). To determine the contribution of the macrophage culturing conditions to this uniform growth/survival defect, we carried out growth curve analyses of each mutant in Dulbecco’s modified Eagle’s medium (DMEM) macrophage medium incubated at 37°C for 48 h. Consistent with the defect observed in macrophages, all three mutant strains displayed growth defects under these culture conditions (Fig. 6B).

**FIG 7:**
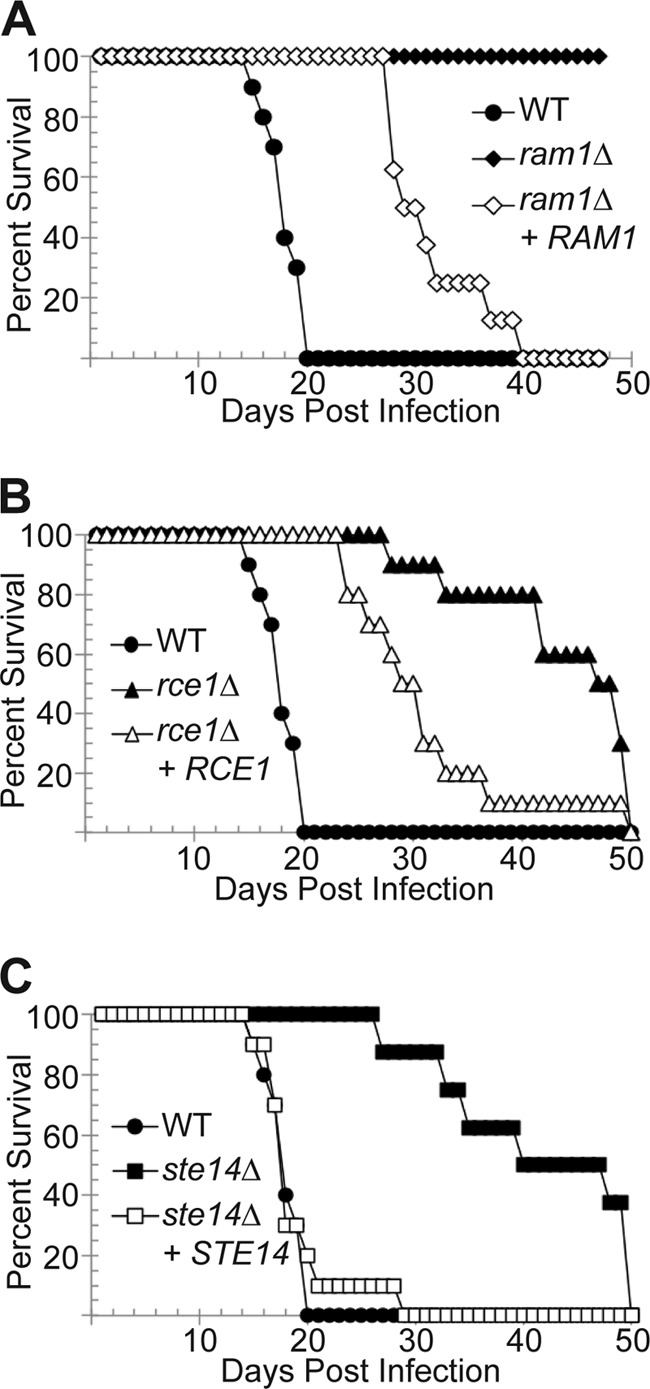
Virulence contribution of farnesylation and postprocessing prenylation enzymes. The *ram1*Δ, *rce1*Δ, and *ste14*Δ mutant strains are attenuated for virulence in a murine model of cryptococcosis. A/Jcr mice were intranasally inoculated with the wild-type (H99), *ram1*Δ (SKE1), *ram1*Δ + *RAM1* reconstituted (SKE26), *rce1*Δ (LKA1), *rce1*Δ + *RCE1* reconstituted (SKE30), *ste14*Δ (YSB1187), and ste14Δ + *STE14* reconstituted (YSB1597) mutant strains. Animal survival was monitored for 40 to 50 days. The statistical significance of differences between survival curves was determined using the log-rank test with Bonferroni’s correction. (A) *P* < 0.001 for all comparisons. (B) *P* < 0.001 for WT versus *rce1*Δ mutant and WT versus *rce1*Δ + *RCE1* reconstituted strain. (C) *P* < 0.001 for WT versus *ste14*Δ mutant and *ste14*Δ mutant versus ste14Δ + *STE14* reconstituted strain.

From these results, we hypothesized that all three strains would display attenuated virulence *in vivo*, and we used a murine inhalation model of cryptococcal infection to directly assess the relative virulence of these strains. We intranasally inoculated 8 to 10 mice with each mutant strain, as well as their respective reconstituted and wild-type strains. During the course of infection, we serially assessed surrogate endpoints of progressive infection known to correlate with impaired survival (weight loss, neurological symptoms, and inability to maintain self-care) (44). Mice infected with the wild-type strain exhibited a median survival time of 18 days. In contrast, all mice infected with the *ram1*Δ strain survived to the end of the observation period (Fig. 7A). Both *rce1*Δ and *ste14*Δ strain-infected mice also exhibited significantly prolonged survival, with median survival times of 48 and 40 days, respectively (Fig. 7B and C). The *ram1*Δ and *rce1*Δ reconstituted strains (SKE26 and SKE30, respectively) resulted in partial restoration of virulence with median survival times of 30 days; the *ste14*Δ reconstituted strain resulted in complete restoration with a median survival time of 18 days (Fig. 7A to C).

To more fully explore the incomplete restoration of virulence in the *ram1*Δ strain background, we generated an additional *ram1*Δ + *RAM1* reconstituted strain (SKE39). This strain was used in all other studies presented here. In contrast to the original *ram1*Δ + *RAM1* (SKE26) strain, this newly created reconstituted strain was able to completely rescue the growth/survival defect of the *ram1*Δ mutant in J774.1 macrophages (see Fig. S1 in the supplemental material). We also confirmed that while the *rce1*Δ + *RCE1* reconstituted strain (SKE30) did not fully restore virulence in the *rce1*Δ strain tested (LKA1 [*rce1*Δ^a^]), an independent *rce1*Δ mutant (SKE51 [*rce1*Δ^b^]) displayed growth/survival defects in J774.1 macrophages that were similar to those of the original mutant strain (see Fig. S2C). These results confirm the role of the Ram1 FTase and the Rce1 and Ste14 postprenylation processing enzymes in supporting full *C. neoformans* virulence, and they also suggest that precise regulation of the expression of prenylation pathway enzymes is important for controlling the complex and composite phenotype of virulence.

10.1128/mSphere.00084-15.1 Figure S1  An independent *ram1*Δ + *RAM1* reconstituted strain fully restores the *in vitro* virulence defects of the *ram1*Δ mutant. The wild type (H99), the *ram1*Δ mutant (SKE1), and a second *ram1*Δ reconstituted strain (*ram1*Δ + *RAM1*^1^; strain SKE39) were each coincubated with activated J774.1 cells for 1 h, followed by removal of nonphagocytosed yeast. Cell survival was assessed at 24 h by quantitative culture. Data are the mean results ± standard errors from 4 replicates. **, *P* < 0.001 (versus the WT and versus the *ram1*Δ + *RAM1*^1^ mutant), as determined by one-way ANOVA and the Tukey-Kramer test. Download Figure S1, PDF file, 0.5 MB. Copyright © 2016 Esher et al. 2016 Esher et al. This content is distributed under the terms of the Creative Commons Attribution 4.0 International license.

10.1128/mSphere.00084-15.2 Figure S2  Two independent *rce1*Δ mutants display similar phenotypes. (A) Overnight cultures of the wild type (H99) and the *rce1*Δ^a^ (LKA1) and *rce1*Δ^b^ (SKE51) mutants were serially diluted, spotted onto YPD medium, and incubated at 30°C, 37°C, and 39°C for 48 h. (B) The wild type (H99) and the *rce1*Δ^a^ (LKA1) and *rce1*Δ^b^ (SKE51) mutants were incubated in YPD liquid medium and shifted to 37°C for 24 h. Cells were imaged with DIC optics to assess temperature-dependent alterations in morphology. Bar, 5 µm. (C) Quantification of morphological defects after 24 h of incubation at 37°C. Data represent the percentages (as means ± standard errors) of budded cells with morphological changes, which included wide bud necks, elongated/unseparated cells, and/or chains of cells. ImageJ software (Fiji) was used to count at least 600 cells over 3 biological replicates for each strain. (WT and *rce1*Δ^a^ strain data also appear in Fig. 3 in the text.) (60). ns, not significant as determined by one-way ANOVA and Tukey’s multiple comparisons test. (D) The wild type (H99) and the *rce1*Δ^a^ (LKA1) and *rce1*Δ^b^ (SKE51) mutants were coincubated with J774.1 cells as described above. *, *P* < 0.05 (versus the WT), as determined by one-way ANOVA and the Tukey-Kramer test. Download Figure S2, PDF file, 1.3 MB. Copyright © 2016 Esher et al. 2016 Esher et al. This content is distributed under the terms of the Creative Commons Attribution 4.0 International license.

## DISCUSSION

**FIG 8:**
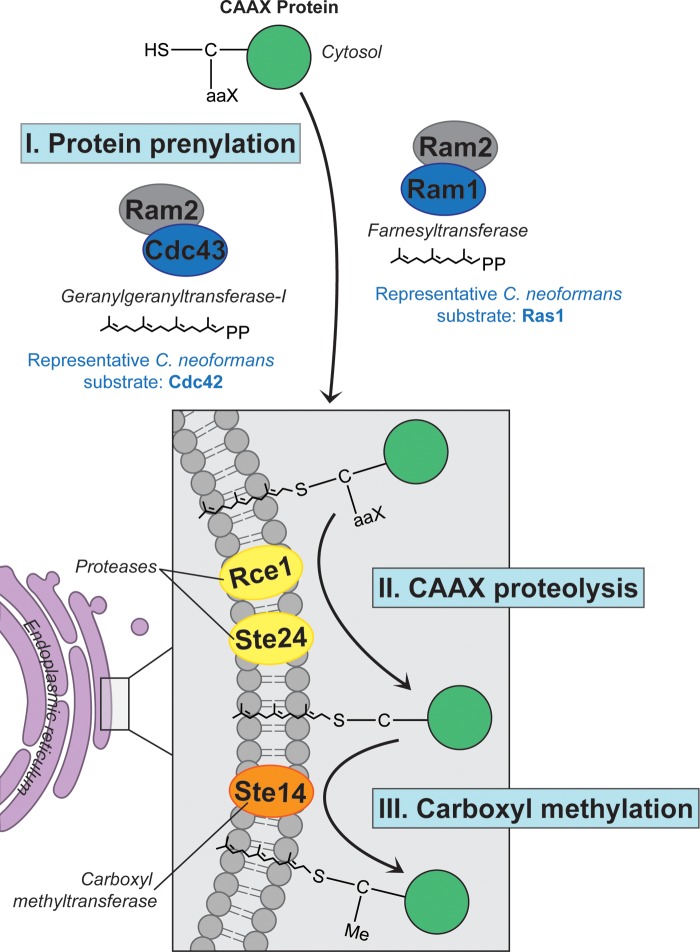
Model of prenylation and postprenylation processing in *C. neoformans*. CAAX proteins are (I) prenylated in the cytosol by the farnesyltransferase or geranylgeranyltransferase enzyme, resulting in initial membrane localization. Next, the prenylated protein undergoes further posttranslational modifications, including (II) cleaving of the C-terminal –AAX by the CAAX proteases Rce1 and Ste24, followed by (III) carboxyl methylation of the terminal cysteine by Ste14, after which they can be trafficked to their destined cellular membrane. In *C. neoformans*, substrate proteins may be trafficked to their destined cellular membrane through pathways independent of the CAAX proteases Rce1 and Ste24 and the carboxylmethyltransferase Ste14.

The protein prenylation pathway is important for the processing and proper function of Ras-like GTPases (Fig. 8). In this study, we have characterized the phenotypic and pathogenesis-relevant consequences for the FTase β-subunit Ram1, as well as the postprenylation processing enzymes in *C. neoformans*.

### *RAM1* is required for substrate protein localization.

Previously, we reported that the *RAM1* gene encoding the FTase β-subunit was essential (35); however, in this study we successfully isolated multiple *ram1*Δ mutant strains using permissive growth conditions and a different, fully virulent, *C. neoformans* strain background. While viable, mutant strains lacking *RAM1* are very temperature sensitive, displaying growth defects at the normally permissive temperature of 30°C. While the *RAM2* gene encoding the prenyltransferase α-subunit is essential in both *S. cerevisiae* and *C. albicans*, the importance of the β-subunits varies among these and other species (26, 38). For example, the genes encoding the GGTase-I β-subunit in *S. cerevisiae* (*CDC43*) and the fission yeast *Schizosaccharomyces pombe* (*cwg1*+) are essential, while the deletion of *CDC43* in *C. albicans* results in a subtle morphology defect (45–48). In contrast, the gene encoding FTase is not essential in *S. cerevisiae* (*RAM1*) or *S. pombe* (*cpp1+*), although the corresponding deletion strains exhibit poor growth at lower temperatures and are unable to grow at 37°C (38, 39).

We also previously demonstrated that the GGTase β-subunit Cdc43 is not essential in *C. neoformans* (8). Predicted targets of GGTase in *C. neoformans* include essential proteins, such as Rho1 (49), suggesting that the FTase and GGTase prenyltransferases have overlapping substrates and compensate for the loss of each other during conditions such as temperature stress. Prenylation via the CAAX motif promotes membrane association of these substrate proteins (15). In *C. neoformans*, mutation of the CAAX motif of either Ras1 or Cdc42 abolishes membrane localization and renders these proteins nonfunctional (8, 9). In contrast, Cdc42 membrane localization is decreased but not abolished in the GGTase *cdc43*Δ mutant strain, suggesting that prenylation by Ram1 is compensating for the loss of Cdc43 function (8). We also find that a similar sharing of substrates may exist for FTase substrates. Ras1 localization to the PM is decreased but not completely abolished in the *ram1*Δ mutant strain. This decrease in Ras1 PM localization occurs in *ram1*Δ mutant cells incubated at 25°C but is more pronounced at higher temperatures. This appears to be a common phenomenon in fungi; *S. cerevisiae* GGTase can compensate for loss of Ram1 at lower temperatures, and *C. albicans* FTase can partially compensate for the loss of Cdc43 (45, 50).

We observed temperature-dependent defects in cytokinesis among our *C. neoformans* strains with mutations in proteins predicted to be involved in prenylation (*ram1*Δ and *cdc43*Δ), prenylation substrates (*cdc42*Δ), and postprenylation processing (*rce1*Δ CAAX protease mutant) (8, 32, 38). However, the *ram1*Δ mutant growth defect exhibited at the normally permissive temperature of 30°C is quite distinct from those observed in *cdc43*Δ, *rce1*Δ, *ras1*Δ, and *cdc42*Δ mutant strains. This suggests that Ram1 has multiple prenylation targets that are important for the growth of *C. neoformans*. The cytokinesis defects observed in the *ram1*Δ mutant strain are likely due in part to the loss of proper Ras1 localization and function. Although the terminal phenotype of the *ras1*Δ mutant strain is characterized by very large unbudded cells, this strain also exhibits a cytokinesis defect that is manifested when cells are initially shifted from 30°C to 37°C (36). Our laboratory previously determined that *C. neoformans* septin localization and function are impaired in *ras1*Δ mutants, contributing to defects in cytokinesis (36). Ras1 mediates cytokinesis by regulating septin function through the activation of Cdc42, which is prenylated by Cdc43 (8). Mutation of any of these downstream targets (Cdc42 and septins, or Cdc43) leads to similar cytokinesis defects in *C. neoformans* (8, 32, 33, 36, 51). Therefore, one would predict that defective prenylation would manifest as a composite phenotype resulting from altered function of many of these downstream target proteins.

### Postprenylation processing enzymes are not individually required for localization of prenylation substrates

Our localization data demonstrate that prenylation is the critical step in determining membrane localization for Ras1 and Cdc42. We were not able to detect changes in membrane localization of Ras1 or Cdc42 in the *rce1*Δ, *ste24*Δ, or *ste14*Δ single mutant strains or in the *rce1Δ ste24*Δ double mutant strain. This is distinct from what has been observed in mammalian cells and *S. cerevisiae*. Both pharmacological and genetic disruption of the *RCE1* gene in both yeast and mammals results in the delocalization of Ras from the PM (52, 53). Disruption of *STE14* in *S. cerevisiae* resulted in a slight decrease in Ras2 membrane localization; however, this did not appear to significantly affect Ras2 function (54). Notably, the disruption of the gene encoding the carboxy methyltransferase in mammalian cells led to the mislocalization of K-Ras (55). These results in mammalian cells have led to an increase in the interest in these downstream processing steps as therapeutic targets, due to the prominent role that Ras proteins play in oncogenesis. However, our data would suggest that the postprenylation processing steps would be less attractive antifungal targets than the prenylation events themselves in terms of specifically interfering with virulence-associated phenotypes and target protein localization.

It is important to note that studies in *S. cerevisiae* have also indicated that the additional M16A metalloproteases Axl1 and Ste23 may contribute to proteolysis of Rce1 and Ste24 substrates in strains lacking both *RCE1* and *STE24* (56). This observation suggests that multiple proteases might share some degree of functional redundancy in CAAX protein modification, perhaps accounting for the more subtle phenotypes in the protease mutants than in the prenyltransferase mutants. It is therefore possible that *C. neoformans* homologs of Axl1, Ste23, or as-yet-uncharacterized proteases similarly contribute to the postprenylation processing of prenylated proteins. The biochemical studies necessary to define the proteolysis and carboxylmethylation states of substrate proteins in *C. neoformans* will be an important future step to confirm the functions of these enzymes.

### Prenylation and postprenylation processing enzymes are required for sexual differentiation in *C. neoformans*

In *C. neoformans*, several important mating determinants have previously been shown to be prenylation substrates. Ras1 alone is required for transcriptional induction of mating pheromone, as well as filamentous differentiation (6, 31). Ras1 also signals downstream to Cdc42 and Rac2 to regulate hyphal transitions that are central to the mating process (7, 28, 32, 33, 36). Additionally, mutational analysis of the Ras1 CAAX motif has demonstrated that Ras-mediated sexual differentiation is dependent on prenylation (9).

To determine which aspects of the prenylation pathway are involved in mating, we examined each mutant strain for unilateral mating defects. We observed that *ram1*Δ and *ste14*Δ strains were completely sterile, with *rce1*Δ displaying patchy, reduced mating structures. The sterility of the *ram1*Δ strain was to be expected considering the delocalization of Ras1 in this strain. However, in the *ste14*Δ and *rce1*Δ mutants, strains in which we did not detect Ras1 delocalization, we still observed mating defects. It is possible that this is the result of subtle decreases in protein membrane localization that we could not detect with our fluorescent protein fusion assays; however, it is more likely due to altered pheromone prenylation. Both *MAT***a** and *MAT*α pheromones are predicted prenylation substrates, and previous studies have indicated that prenylation defects can affect pheromone function. In fact, an **a**-mating pheromone precursor is one of the only known substrates of the alternative Ste24 protease in *S. cerevisiae* (19). While the *rce1*Δ mutant displays a modest mating defect, the *rce1Δ ste24*Δ double mutant strain is completely sterile, suggesting that the sterility of these strains is due to lack of pheromone processing. Interestingly, a *MAT*α *rce1*Δ mutant strain crossed to a *MAT***a** *ste24*Δ mutant strain also displays decreased mating, suggesting that Rce1 and Ste24 share overlapping but dosage-dependent roles in the postprenylation processing of pheromone.

### Prenylation and postprenylation processing enzymes impact pathogenesis

Our virulence studies offer both expected results and some that could not have been readily predicted by *in vitro* surrogate markers of microbial fitness. For example, it was not surprising that the *ram1*Δ mutant has a defect in virulence in an animal model of systemic cryptococcosis due to its temperature sensitivity and cytokinesis defects. Since the *rce1*Δ mutant strain also exhibits a more subtle temperature sensitivity, it is to be expected that this mutant strain also demonstrates a partial reduction in virulence. In contrast, the *ste14*Δ mutant displays a reduction in virulence despite lacking *in vitro* phenotypes typically associated with decreased pathogenesis. Due to its role in pheromone processing, the *ste14*Δ mutant strain does exhibit a mating defect; however, loss of pheromone does not significantly affect *C. neoformans* pathogenesis (57). It is more likely that the virulence defect of the *ste14*Δ mutant, and perhaps the *rce1*Δ mutant as well, may be due to loss of processing of other targets, such as *C. neoformans* Rho proteins, as has been suggested in mammalian systems (58). The primary *in vitro* phenotypes that correlate with altered fitness *in vivo* were subtle thermotolerance defects at 39°C. This also potentially suggests the possibility that there are host-specific stresses that are not well mimicked by standard *in vitro* growth conditions, confirming the necessity to assess virulence in more relevant models.

In summary, these studies have confirmed the central role of prenylation enzymes in the growth, differentiation, and virulence potential of an important fungal pathogen. This process is mediated by the regulation of target membrane localization. We have found that the enzymes that mediate postprenylation processing events are not required for the membrane localization of the target proteins tested here. This observation is surprising, since CAAX proteolysis and carboxyl methylation are often needed in other organisms for the full maturation of both farnesylated and geranylgeranylated proteins. These postprenylation processing enzymes were still required for full virulence, indicating the possibility of additional, undefined targets of these proteins. The relative roles of the enzymes functioning in this process in *C. neoformans* are demonstrated in a model in Fig. 8, in which prenylation, but not postprenylation processing, is required for membrane localization of target proteins in *C. neoformans*. Our studies emphasize the importance of prenylation and its target proteins in the response to cell stress, especially that encountered by microbial pathogens within the infected host.

## MATERIALS AND METHODS

### Strains, media, and growth conditions.

All *Cryptococcus neoformans* strains used in this study are listed in Table 1. Unless otherwise stated, all strains were generated in the *C. neoformans* var. *grubii* strain H99 background. Strains were maintained on YPD medium (2% yeast extract, 1% peptone, 2% dextrose). Mating experiments were carried out by coculturing strains of opposite mating types on Murashige and Skoog (MS) mating medium (Sigma) (59) in the dark at 25°C and photographed after 7 days (59). Morphology and localization images were obtained by incubating strains overnight in liquid YPD medium at 25°C with aeration, diluting into fresh YPD, and incubating for 4 or 24 h, as specified in the figure legends, at 25°C or 37°C with aeration prior to imaging. Quantifications of cells with morphological defects were carried out using the cell counter feature in ImageJ (Fiji) software (60). Temperature sensitivity was determined by incubating cultures overnight in liquid YPD medium at 25°C with aeration, serially diluting into fresh YPD, spotting onto YPD plates in 5-µl aliquots, and incubating at 25°C, 30°C, or 37°C. Plates were photographed after 48 h of incubation. For growth curve analysis, cells were incubated overnight in liquid YPD at 30°C with aeration and diluted into Dulbecco’s modified Eagle’s medium (DMEM), and 200 µl of each strain was plated in quadruplicate into a 96-well plate. Plates were shaken at 37°C with aeration in a FLUOstar Optima microplate reader (BMG Labtech) for 48 h, and absorbance readings were taken every hour.

**TABLE 1:** *C. neoformans* strains used in this study

Strain	Genotype	Source or reference
H99	*MAT*α	67
KN99**a**	*MAT***a**	68
JLCN437	*MAT*α *cku80*::*NEO*	37
KS68-2	*MAT*α *cku80*::*NEO ram1*::*NAT*	This study
SKE1	*MAT*α *ram1*::*NEO*	This study
SKE26	*MAT*α *ram1*::*NEO RAM1-NAT*	This study
SKE39	*MAT*α *ram1*::*NEO RAM1-NAT*	This study
LKA1 (*rce1*Δ^a^)	*MAT*α *rce1*::*NEO*	This study
SKE51 (*rce1*Δ^b^)	*MAT*α *rce1*::*NEO*	This study
SKE30	*MAT*α *rce1*::*NEO RCE1-NAT*	This study
LKA14	*MAT***a** *ste24*::*HYG*	This study
YSB1187	*MAT*α *ste14*::*NAT*	This study
YSB1597	*MAT*α *ste14*::*NAT+ste14-NEO*	This study
LKA18	*MAT*α *rce1*::*NEO ste24*::*HYG*	This study
SKE32	*MAT*α *rce1*::*NEO ste24*::*HYG+RCE1-NAT*	This study
CBN121	*MAT*α*mCh-RAS1-NAT*	This study
CBN302	*MAT*α *GFP-CDC42-NAT*	This study
SKE17	*MAT*α *ram1*::*NEO mCh-RAS1-NAT*	This study
SKE19	*MAT*α *ram1*::*NEO GFP-CDC42-NAT*	This study
LKA7	*MAT*α *rce1*::*NEO mCh-RAS1-NAT*	This study
SKE15	*MAT*α *rce1*::*NEO GFP-CDC42-NAT*	This study
CBN308	*MAT*α *ste14*::*NAT mCh-RAS1-NEO*	This study
SKE53	*MAT*α *ste14*::*NAT GFP-CDC42-NEO*	This study
SKE22	*MAT*α *rce1*::*NEO ste24*::*HYG GFP-CDC42-NAT*	This study
CBN453	*MAT*α *rce1*::*NEO ste24*::*HYG mCh-RAS1-NAT*	This study

### Genetic manipulation of *C. neoformans.*

*C. neoformans* targeted gene deletion mutants were generated by homologous recombination to replace the open reading frame (ORF) of each gene with a mutant allele containing a dominant selectable marker constructed by three-piece PCR overlap extension (61). The *RAM1* ORF was replaced with the nourseothricin (*NAT*) cassette (62) in the *cku80* mutant strain and was also replaced with the neomycin (*NEO*) cassette (40) in the wild-type strain H99, generating strains KS68-2 and SKE1, respectively. The ORF for *RCE1* was replaced with the *NEO* cassette, generating strain LKA1. The *STE14* ORF was replaced with the *NAT* cassette, generating strain YSB1187. The ORF for *STE24* was replaced with the hygromycin (*HYG*) cassette (63), generating strain LKA14. The *rce1Δ ste24*Δ double mutant strain (LKA18) was generated by deleting the *STE24* ORF in strain LKA1. Genomic integration and homologous recombination were achieved by biolistic transformation (64). Deletion and reconstitution of each mutant were confirmed by PCR and Southern blot analysis. Reconstitution strains of the *ram1*Δ, *rce1*Δ, and *ste14*Δ mutant strains were generated by reintroduction of the wild-type gene by biolistic transformation. We also generated a second, independent, *rce1*Δ mutant (SKE51 [*rce1*Δ^b^]). This mutant displayed phenotypes similar to those of the original *rce1*Δ mutant (LKA1 [*rce1*Δ^a^]), including a growth defect at 39°C (manifested as smaller colony size than for the WT), similar morphological changes, and a significant defect in growth/survival within J774.1 macrophages (see Fig. S2 in the supplemental material).

Plasmids expressing the fluorescent fusion protein-encoding genes *mCherry-RAS1* and *GFP-CDC42* (8), using the constitutive *HIS3* promoter, were introduced into wild-type H99 and *ram1*Δ, *rce1*Δ, *ste14*Δ, and *rce1Δ ste24*Δ mutant strains by biolistic transformation (64).

### Microscopy.

Differential interference microscopy (DIC) and fluorescent images were visualized with a Zeiss Axio Imager.A1 fluorescence microscope (100× objective) using the appropriate filter set. Images were captured with an AxioCam MRm digital camera and processed using ZEN Pro software (Zeiss). High-resolution fluorescent images were visualized using a DeltaVision Elite deconvolution microscope. Images were captured by a Coolsnap HQ2 high-resolution charge-coupled-device (CCD) camera and processed using softWoRx software (GE). Images being directly compared were taken with identical exposures. Images were additionally analyzed using ImageJ (Fiji) software (60).

### Cell fractionation and Western blot analysis.

To determine the proportions of Ras1 localized in cell membrane versus soluble fractions in wild-type and mutant strains, we performed subcellular fractionation. Cells were inoculated into liquid YPD medium with shaking for 16 to 18 h at 25°C. Quadruplicate samples (20 ml, normalized to an optical density [OD] of 1.0) for each strain were centrifuged (3,000 rpm for 15 min), rinsed twice in water, and resuspended in 1 ml of lysis buffer (10 mM Tris-HCl, 150 mM NaCl, 0.5 mM EDTA, 1× protease inhibitors [Complete mini, EDTA-free; Roche], 1× phosphatase inhibitors [Phos-stop; Roche], and 1 mM phenylmethylsulfonyl fluoride [PMSF]). Each sample was pelleted, decanted, and flash frozen on dry ice. Lysis was performed by adding 0.5 ml of glass beads to each sample and bead beating (Mini-BeadBeater-16 [BioSpec]) for 30 s with 1 min of incubation on ice for 6 to 8 cycles. Following lysis, the crude lysate was collected from the pellet by adding 0.4 ml of lysis buffer, centrifuging to pellet cell debris, and transferring the lysate to a fresh tube (repeated 2 times). Like samples were combined and diluted to 5 ml with lysis buffer. To separate the soluble and insoluble fractions, crude lysates were separated by ultracentrifugation (100,000 × *g*) for 1 h at 4°C. The soluble fraction was transferred to a new tube and the insoluble pellet fraction was resuspended in the equivalent volume of lysis buffer containing 1% Triton X-100.

For Western blot analysis, samples were normalized by total protein concentration in the crude lysate, diluted in 4× lithium dodecyl sulfate (LDS) loading sample buffer, and boiled for 5 min. Fifteen microliters of each sample was loaded and separated by electrophoresis on a NuPAGE 4 to 12% bis-Tris gel (Invitrogen) with morpholinepropanesulfonic acid (MOPS) buffer. Samples were transferred to Invitrolon polyvinylidene difluoride (PVDF) membrane (Invitrogen) and blocked by incubating in Starting Block T20 (Pierce) for 1 h. To detect mCherry-labeled proteins, blots were incubated with an anti-red fluorescent protein (RFP) primary antibody (1:5,000 dilution, Clontech) overnight. Blots were washed 3 times for 10 min with TBST (Tris-buffered saline with Triton X-100), incubated with a peroxidase-conjugated secondary antibody (1:50,000 dilution of anti-rabbit antibody; Jackson Laboratory) for 1 h, and washed 3 times for 10 min with TBST. Proteins were detected by enhanced chemiluminescence (ECL Prime Western blotting detection reagent; GE Healthcare).

### Macrophage and animal experiments.

Survival within macrophages was tested by coculturing *C. neoformans* cells with J774.1 murine macrophagelike cells (43). Briefly, 1 × 10^5^ J774.1 cells in DMEM were added per well in a 96-well plate and allowed to adhere overnight at 37°C with 5% CO_2_. J774.1 cells were activated by incubating with 10 nM phorbol myristate acetate (PMA) for 1 h at 37°C with 5% CO_2_ or by adding gamma interferon (IFN-γ; 100 µg/ml) and lipopolysaccharide (0.6 µg/ml) and incubating overnight at 37°C with 5% CO_2_. *C. neoformans* cultures (wild-type strain H99 and *ram1*Δ, *rce1*Δ, and *ste14*Δ mutant strains) were incubated overnight in YPD medium with shaking, pelleted by centrifugation, washed twice with phosphate-buffered saline (PBS), and opsonized by adding DMEM containing 1 µg/ml monoclonal antibody (MAb) 18B7 for 1 h at 37°C (65, 66). Opsonized cells (1 × 10^5^) were added to each well of activated J774.1 cells and coincubated for 1 h. Nonphagocytosed *C. neoformans* cells were removed by washing 3 times with PBS. Fresh DMEM was added (200 µl per well), and the plates containing the cocultured cells were incubated for 24 h at 37°C with 5% CO_2_. To release the phagocytosed *C. neoformans* cells from the J774.1 cells, sterile water was added to each well, pipetted vigorously, and transferred to a fresh tube. This was repeated twice. Extracted cells were serially diluted and plated onto YPD agar to assess the number of viable *C. neoformans* cells by quantitative culture. The statistical significance of the growth and survival of each mutant strain compared to those of the WT was determined using one-way analysis of variance (ANOVA) and the Tukey-Kramer test (JMP software; SAS Institute, Cary, NC).

Virulence was assessed by using the murine inhalation model of systemic cryptococcosis as described previously (44). In brief, groups of 10 female A/Jcr mice were anesthetized with isoflurane and then intranasally inoculated with 5 × 10^5^ *C. neoformans* cells of the following strains: wild type (H99) and *ram1*Δ (SKE1), *ram1*Δ + *RAM1* (SKE26), *rce1*Δ (LKA1), *rce1*Δ + *RCE1* (SKE30), *ste14*Δ (YSB1187), and *ste14Δ + STE14* (YSB1189) mutants. The mice were monitored and sacrificed based on predetermined clinical endpoints that predict imminent mortality. The statistical significance of differences between survival curves of all animals infected with each strain was determined using the log-rank test with Bonferroni correction (JMP software; SAS Institute, Cary, NC). All studies were performed in compliance with Duke University institutional guidelines for animal experimentation.
