# Supplementary material for: Relative Contributions of Prenylation and Postprenylation Processing in Cryptococcus neoformans Pathogenesis
Source: mSphere. 2016 Mar 30;1(2):e00084-15. doi: 10.1128/mSphere.00084-15 (PMC4894686; doi:10.1128/mSphere.00084-15)
Supplement: Figure S1 [file sph002162047sf1.pdf]

A

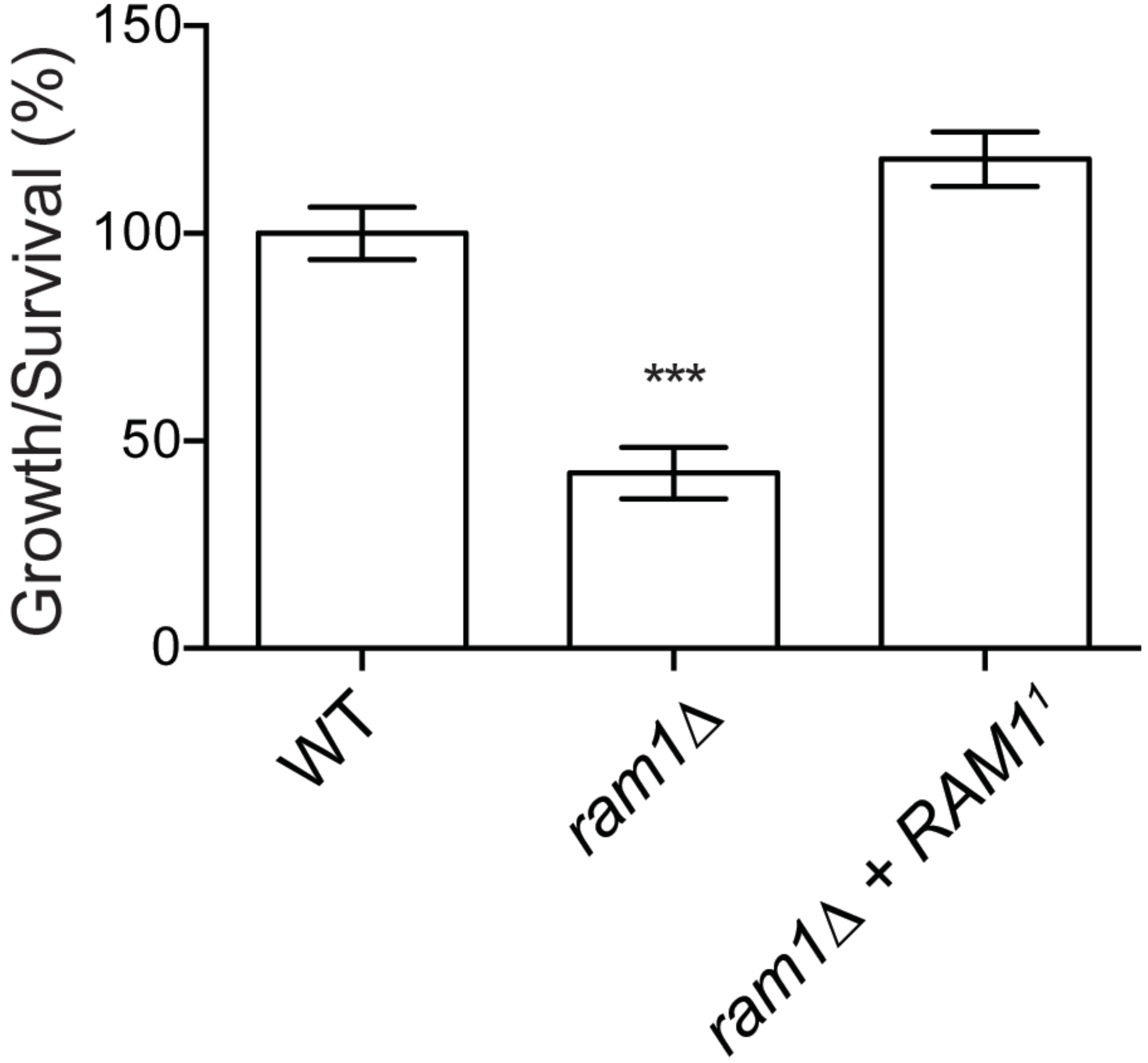

Figure S1. An independent ram1Δ + RAM1 reconstituted strain fully restores ram1Δ mutant in vitro virulence defects. Wild type (H99), ram1Δ (SKE1), and a second ram1Δ reconstituted strain (ram1Δ + RAM11, SKE39) were each co-incubated with activated J774.1 cells for 1 h, followed by removal of non-phagocytosed yeast. Cell survival was assessed at 24 h by quantitative culture. Data are represented as mean ± standard error of 4 replicates. **, P < 0.001 (vs. WT and vs. ram1Δ + RAM11), as determined by one-way ANOVA and Tukey-Kramer.
