# Supplementary material for: Relative Contributions of Prenylation and Postprenylation Processing in Cryptococcus neoformans Pathogenesis
Source: mSphere. 2016 Mar 30;1(2):e00084-15. doi: 10.1128/mSphere.00084-15 (PMC4894686; doi:10.1128/mSphere.00084-15)
Supplement: Figure S2 [file sph002162047sf2.pdf]

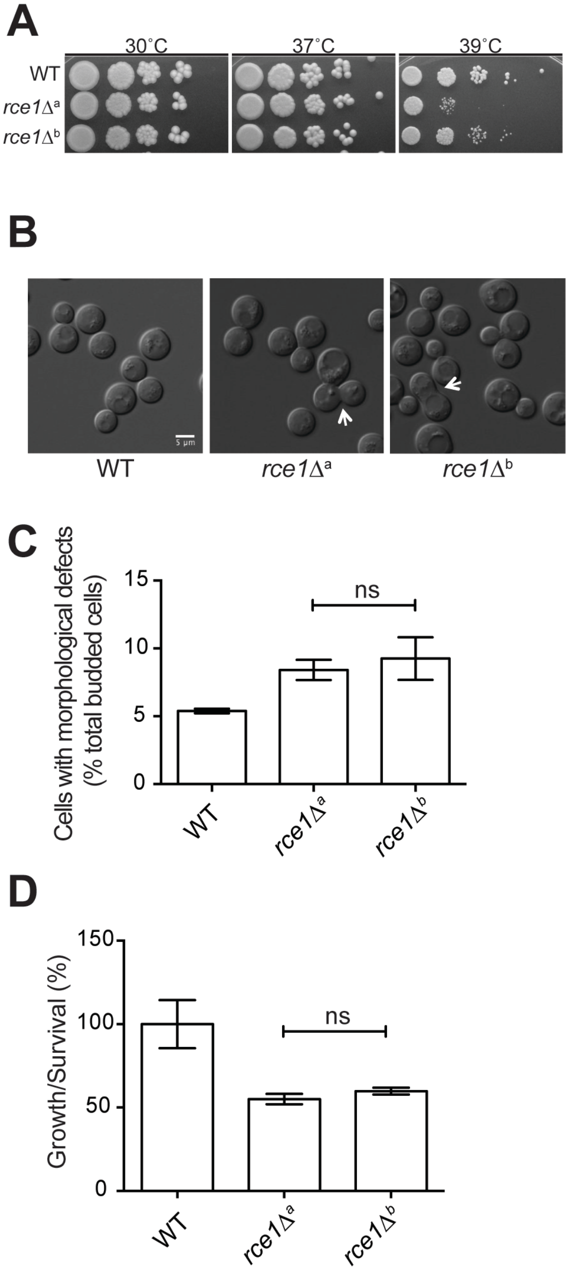

Figure S2. Two indepdent rce1Δ mutants display similar phenotypes. (A) Overnight cultures of wild type (H99), rce1Δa (LKA1), and rce1Δb (SKE51) were serially diluted, spotted onto YPD medium, and incubated at 30˚C, 37˚C, and 39˚C for 48 h. (B) Wild type (H99), rce1Δa (LKA1), and rce1Δb (SKE51) were incubated in YPD liquid medium and shifted to 37˚C for 24 h. Cells were imaged with DIC optics to assess temperature-dependent alterations in morphology. Bar, 5 µm. (C) Quantification of morphological defects after 24 h incubation at 37˚C. Data represent mean ± standard error percent of budded cells with morphological changes, which included wide bud necks, elongated/unseparated cells, and/or chains of cells. ImageJ (Fiji) software was used to count at least 600 cells over 3 biological replicates for each strain (WT and rce1Δa data also appear in Figure 3) (add citation). ns, not significant as deteremined by one-way ANOVA and Tukey multiple comparisons test. (D) Wild type (H99), rce1Δa (LKA1), and rce1Δb (SKE51) were co-incubated with J774.1 cells as decribed above. *, $P < 0.05$ vs. WT, as determined by one-way ANOVA and Tukey-Kramer.
